# Hazardous Hernia: Mechanical Ventilation in an Adult With a Congenital Diaphragmatic Hernia

**DOI:** 10.7759/cureus.110977

**Published:** 2026-06-16

**Authors:** Julie Gaudin, Sneh Parekh, Claudia Aleman Oliva, Austin Haley, Pankit Patel

**Affiliations:** 1 Internal Medicine, University of Florida College of Medicine – Jacksonville, Jacksonville, USA; 2 Pulmonary, Critical Care, and Sleep Medicine, University of Florida College of Medicine - Jacksonville, Jacksonville, USA

**Keywords:** adult congenital diaphragmatic hernia, diaphragmatic hernias, gastric insufflation, hypoxic respiratory failure, mechanical ventilation, prolonged mechanical ventilation, pulmonary hypoplasia

## Abstract

Congenital diaphragmatic hernias (CDHs) are developmental defects often found in infancy; however, they may be rarely found as a delayed diagnosis in adults. Although often an incidental finding, adult patients with preexisting CDH who present with symptoms of respiratory distress pose unique challenges in airway management. Complications include gastric insufflation, mechanical compression of the surrounding viscera, exacerbation of respiratory distress, and failed ventilatory weaning. We present the case of a 70-year-old female with acute hypoxic respiratory failure requiring mechanical ventilation in the setting of a large, preexisting CDH. The patient’s hospital course was complicated by gastric insufflation requiring gastric decompression, with the need for prolonged ventilator support and repeated extubation failure requiring tracheostomy placement. The literature on ventilator management in adults with CDH remains limited; however, lung-protective ventilation is recommended due to the risk of gastric insufflation and mediastinal compression due to preexisting CDH.

## Introduction

Congenital diaphragmatic hernias (CDHs) are seen in about 1 to 4 individuals per 10,000 births [1]. They are caused by a developmental defect of the diaphragm that leads to herniation of the abdominal organs into the thoracic cavity, which can result in pulmonary hypoplasia and abnormal development of the pulmonary vasculature due to mass effect and mechanical compression [2,3]. CDHs are categorized into three different types based on the location of the diaphragmatic defect. Bochdalek hernias are the most common type and are caused by a defect in the posterolateral portion of the diaphragm and are predominantly left-sided [2]. Morgagni hernias are caused by anteromedial diaphragmatic defects [4,5]. Finally, hiatal hernias result from protrusion of the abdominal contents through the diaphragm hiatus [4,5]. Although congenital hernias are usually found during infancy or earlier in life, there are few cases with delayed diagnosis into adulthood. Acquired diaphragmatic hernias are diaphragmatic defects that typically occur in adults due to physical strain, prior surgery, or blunt or penetrating trauma, including falls. Surgical repair is typically recommended once diagnosed to prevent further complications. In cases of delayed diagnosis into late adulthood, individuals may present with mild dyspnea or dysphagia, while more severe cases may involve obstruction, incarceration, or strangulation of the herniated viscera [5]. Patients with a preexisting CDH who present with symptoms of respiratory distress pose unique challenges pertaining to airway management and ventilatory support. Certain risks, including gastric insufflation and worsening herniation, must be considered, as they can otherwise predispose to respiratory failure. We present the case of a 70-year-old female with a preexisting history of a large CDH without a history of surgical repair or a recent ground-level fall, who presented with acute hypoxic respiratory failure and aspiration requiring multiple intubations and eventual tracheostomy, along with a discussion on management considerations.

## Case presentation

A 70-year-old female with a past medical history including a CDH, prior cerebrovascular accident, gastroesophageal reflux disease, and recent left humerus periprosthetic fracture due to a ground-level fall presented after being found unresponsive at a rehabilitation facility. She was intubated before presentation in the setting of acute hypoxic respiratory failure and acute encephalopathy, with her last known normal three hours before arrival at the emergency department. Vital signs were notable for hypothermia to 33.8°C (reference range: 36.5-37.3°C). Chest radiograph (CXR) revealed placement of the endotracheal tube terminating 1.8 cm from the carina, with presentation of a large diaphragmatic hernia causing mass effect on the mediastinum and the right lung (Figure 1).

**Figure 1:**
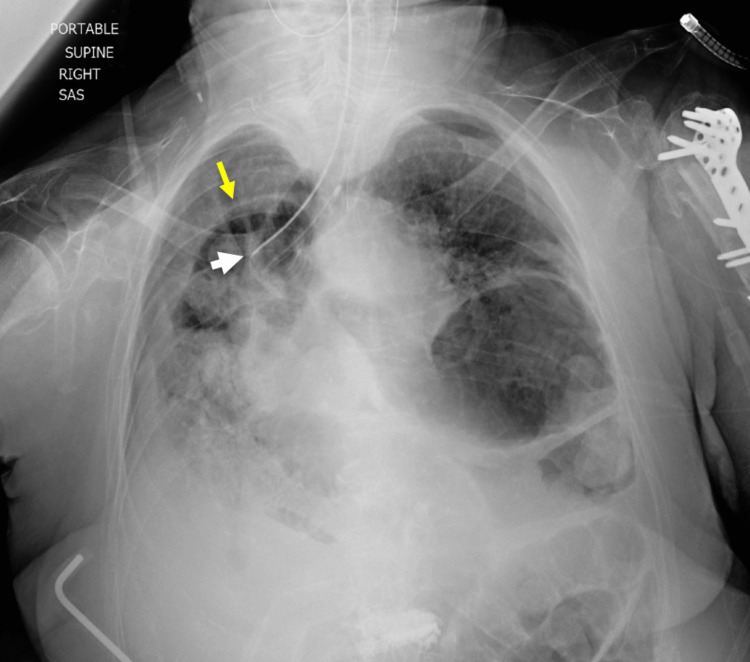
Chest radiograph with the presence of a large diaphragmatic hernia (yellow arrow) with a mass effect on the mediastinum and right lung. The tip of the endotracheal tube (white arrow) is seen terminating 1.8 cm from the carina, displaced secondary to the preexisting large diaphragmatic hernia.

A computed tomography (CT) of the head revealed no evidence of an acute infarct. Further imaging studies with a CT angiography of the chest, abdomen, and pelvis showed consolidation within the left lower lobe of the lung with concern for pneumonia and a large diaphragmatic hernia containing distended bowel loops with protrusion into the right lung field (Figures 2A, 2B).

**Figure 2:**
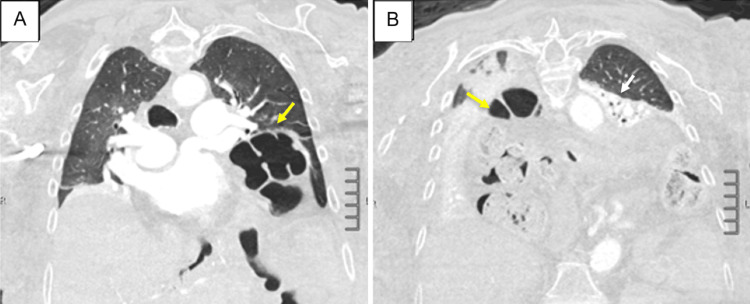
(A, B) Computed tomography angiography of the chest, abdomen, and pelvis showing continued demonstration of the large diaphragmatic hernia with protrusion into the right and left lung fields (yellow arrows). The left lung field with superimposed consolidation (white arrow).

The patient was started on intravenous antibiotics with vancomycin and piperacillin-tazobactam for the left lower lobe pneumonia before deescalating to amoxicillin-clavulanate to complete a seven-day course.

Our patient’s oxygenation and mental status improved, and she was extubated to a nasal cannula on day four of the hospitalization. However, on day six, the patient became tachypneic with a respiratory rate of 30 breaths per minute, with associated hypoxia requiring an increase from 2 L to 6 L of supplemental nasal cannula. The patient subsequently had altered mentation, bradycardia, and hypotension along with the acute hypoxic respiratory failure, prompting reintubation. CXR demonstrated bilateral pleural effusions, worse on the right side, with redemonstration of a large diaphragmatic hernia (Figure 3).

**Figure 3:**
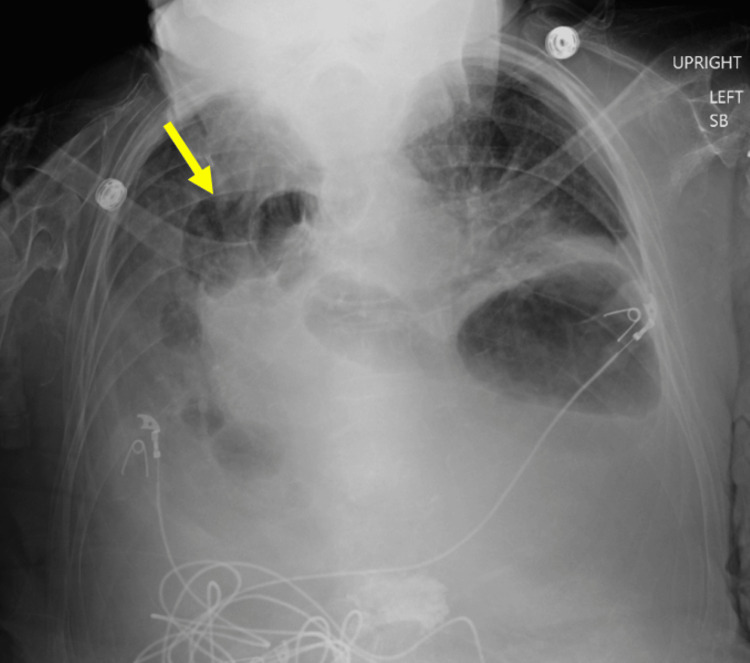
Chest radiograph with bilateral pleural effusions, worse on the right side and redemonstration of a large diaphragmatic hernia (yellow arrow).

The patient was eventually extubated again on day eight of the hospitalization; however, she required reintubation on day nine following an aspiration event. The ventilator settings were set to pressure-regulated volume control with a tidal volume of 380 mL (approximately 7 mL/kg of ideal body weight of 55 kg), a respiratory rate of 15 breaths per minute, a positive end-expiratory pressure (PEEP) of 8 cm H_2_O, and fraction of inspired oxygen (FiO_2_) of 40%. CXR following reintubation showed worsening aeration within the bilateral lung fields, worse on the right, with bilateral pleural effusions and bibasilar passive atelectasis (Figure 4).

**Figure 4:**
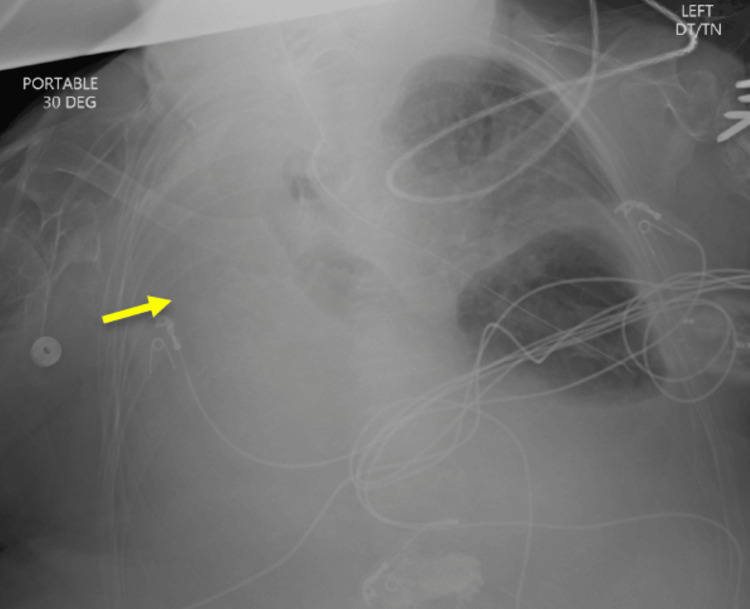
Chest radiograph following reintubation and aspiration event on day nine of hospitalization with bilateral pleural effusions and atelectasis, appearing worse in the right lung (yellow arrow) compared to the left.

Her hospital course was further complicated by gastric insufflation demonstrated on CXR on day 16 of hospitalization requiring nasogastric tube placement during positive-pressure ventilation with minimal improvement in ventilator requirements (Figure 5).

**Figure 5:**
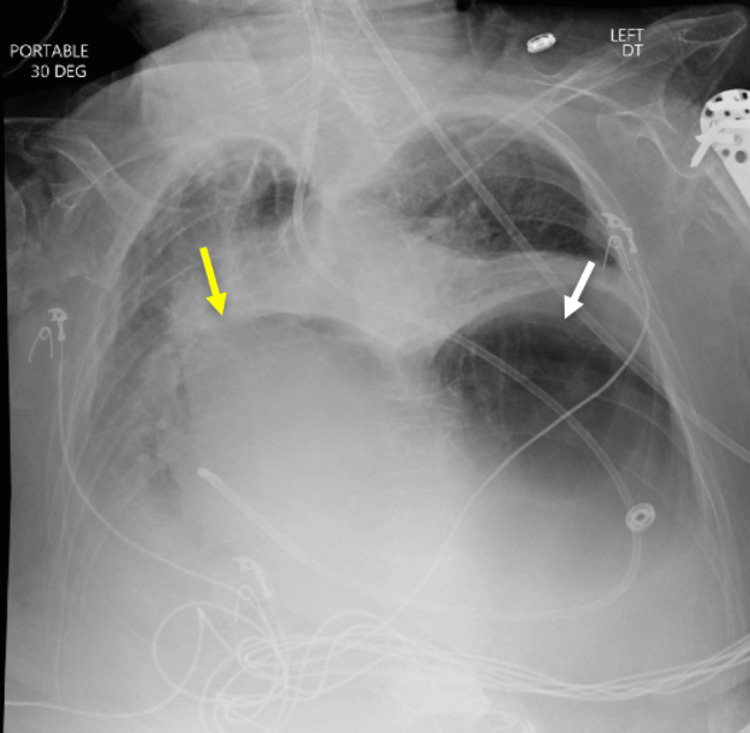
Chest radiograph with interim gaseous distention (white arrow) of the herniated stomach and/or large bowel, measuring up to 25 cm in the transverse dimension, with resultant severe mass effect upon the heart and mediastinum. Redemonstration of large diaphragmatic hernia with displacement of the right diaphragm and mass effect (yellow arrow).

In the setting of prolonged mechanical ventilation and development of critical illness myopathy, the patient continued to have difficulty tolerating pressure support trials at pressure support of 10 cm H_2_O with PEEP of 6 cm H_2_O and FiO_2_ of 40%. A bedside percutaneous tracheostomy was performed, and the patient was eventually discharged to a long-term care facility for continued ventilator management.

## Discussion

CDHs are usually discovered early in life, often during infancy. There have been a few cases found in adulthood as incidental findings with non-specific symptoms, such as abdominal pain, nausea, vomiting, cough, or dyspnea [2,4]. Acquired diaphragmatic hernias typically occur secondary to falls, physical strain, or blunt trauma. Although our patient did have a preexisting CDH which was asymptomatic, she suffered a recent ground-level fall which may have caused increased herniation leading to the presentation of acute hypoxic respiratory failure and respiratory distress. Once identified, surgical repair is definitively recommended to prevent future complications including incarceration or strangulation of the hernia [5]. In our patient’s case, surgical repair was offered before hospitalization; however, it was not pursued. During hospitalization, acute surgical repair was deferred due to clinical instability.

Rare cases of CHDs may present in adulthood with symptoms of respiratory distress, posing unique challenges in ventilatory management due to underlying cardiopulmonary dysfunction and mass effect of hernia contents. Non-invasive positive pressure ventilation and invasive ventilatory support may increase herniation due to worsening of gastric insufflation, resulting in mediastinal shift and mechanical compression of the surrounding structures along with exacerbation of respiratory distress. Gastric decompression may be beneficial to reduce gaseous distension; however, in our patient’s case, prolonged ventilatory support was still required. Furthermore, the presence of a CDH may lead to difficulty in ventilatory weaning due to complications, such as lung atelectasis, pneumothorax, aspiration, or gastric obstruction [6-8]. The literature on respiratory distress and ventilatory support in adults with CDH remains limited, necessitating case-specific and provider-dependent management.

CDHs are associated with pulmonary hypoplasia due to abnormal development of the pulmonary vasculature secondary to mechanical compression from hernia contents. This defect in the development of lung tissue makes the lungs highly susceptible to barotrauma and volutrauma. As such, lung-protective ventilation remains important when mechanical ventilation is required in such patients [9]. These include limiting peak inspiratory pressures to ≤25 cm H_2_O, using smaller tidal volumes, and allowing for permissive hypercapnia (PaCO_2_ below 60-70 mmHg and pH above 7.2-7.25) [9]. Other techniques involving high-frequency oscillatory ventilation can also be used, with some studies showing improved patient outcomes; however, the overall literature shows conflicting results. Underlying cardiopulmonary dysfunction, chronic lung hypoplasia, and obstructive airway disease are additionally seen in adult patients with CDH. As such, there is significant risk for ventilatory-perfusion mismatching, prolonged ventilatory weaning, and higher risk for extubation failure. This was directly observed in our patient with aspiration events and failed extubation, resulting in the need for tracheostomy placement.

## Conclusions

CDHs are developmental defects that can present with significant respiratory compromise due to the anatomic displacement of abdominal contents into the thoracic cavity. In the developmental phase, pulmonary hypoplasia is a known consequence resulting in the development of weaker lungs, more prone to injury from specific insults. CDHs often present in infancy with signs of respiratory distress, prompting early surgical repair. Delayed diagnosis into adulthood is rare. In adults presenting with respiratory distress with a known history of CDH, lung-protective ventilation remains important, as positive pressure ventilation may worsen gastric insufflation and mediastinal compression. Additionally, weaning ventilator support in these patients is a challenge, and early tracheostomy placement must be considered to assist with ventilatory liberation.
